# Experimental Verification of* CAPN1* and* CAST* Gene Polymorphisms in Different Generations of Da-Heng Broilers

**DOI:** 10.1155/2017/7968450

**Published:** 2017-06-21

**Authors:** Yu-Guang Zhou, Yong Xiong, Chao-Wu Yang, Xiao-Song Jiang, Jin-Shan Ran, Jie Jin, Ye Wang, Dan Lan, Peng Ren, Yao-Dong Hu, Yi-Ping Liu

**Affiliations:** ^1^Farm Animal Genetic Resources Exploration and Innovation Key Laboratory of Sichuan Province, Sichuan Agricultural University, Chengdu Campus, Chengdu 611130, China; ^2^Sichuan Animal Science Academy, Chengdu 610066, China

## Abstract

The micromolar calcium activated neutral protease* (CAPN1)* and calpastatin* (CAST)* have been widely regarded as genes related to muscle growth and meat tenderness. The objective of this study was to verify the association of SNPs of* CAPN1* and* CAST *genes with carcass and tenderness traits and search the possible change patterns of SNPs in* CAPN1* and* CAST* genes in six generations of broiler breeding process for growth rate, efficiency, and reproduction, during the third generation and the ninth generation, respectively. We found that, for* CAPN1*, genetic effects between SNPs (G3535A, C7198A) and meat tenderness were similar in different generations, while SNP3 (G7324A) was a novel polymorphism and had significant association with carcass and tenderness traits (*P* < 0.05) in this study. Furthermore, there was significant association between SNP4 (G9950A) and carcass indexes instead of tenderness traits (*P* < 0.05) which was consistent in the two generations. Moreover, although SNP6 (G37868A) of* CAST *had no relevance to carcass traits or tenderness traits in the third generation, it showed significant association with LW and CW in the ninth generation (*P* < 0.05).

## 1. Introduction 

Carcass traits can intuitively reflect the production of broilers. Color, drip loss, juiciness, tenderness, and flavor are the major traits for chickens, among which tenderness can directly affect the taste of chicken [1]. However, tenderness is a complex trait in breeding programs for its complex indexes and factors [2]. With the development of molecular technology, molecular markers have a good theoretical basis in correlating with production traits and improve the breeding process [3].

In the past two decades, genetic markers related to production traits and tenderness traits have been largely reported [4–6]. Among these genes, calpain systen has been widely reported to be related to postmortem muscle proteolysis and tenderization [7, 8]. Calpain is a ubiquitous cytoplasmic cysteine protease requiring calcium ions for activity [9]. Calpain system consists of the ubiquitously expressed *μ*-calpain (CAPN1) and m-calpain (CAPN2) and the only endogenous inhibitor of CAPN1 and CAPN2-calpastatin (CAST) [10]. Excitation of CAPN1 needs calcium at micromolar concentrations and CAPN2 requires calcium for activity at millimolar concentrations [11].* CAPN1* gene encodes the large subunit of *μ*-calpain and the* CAST* gene encodes inhibitor of the calpains [12]. CAPN1 gene is located in chicken chromosome 3 with 14200 bp in length and contains 21 exons and 20 introns, while CAST gene consisting of 31 exons and 30 introns is mapped to chromosome Z, with 61188 bp in length.

Currently, multiple polymorphisms of genes in calpain system have been identified as potentially relevant to meat quality traits. Of these,* CAPN1* has a significant association with postmortem proteolysis and meat tenderization [13, 14], while* CAPN2* was thought to play a marginal role [15]. Two nonsynonymous single nucleotide polymorphisms (SNPs) of* CAPN1* gene, C316G and A530G, have been found to be associated with meat quality traits in cattle [16–19]. At the same time C4751T in* CAPN1* has a significant association with shear force at 7, 14, and 21 days of postmortem [20]. In chickens, four SNPs (C2546T, G3535A, C7198A, and G9950A) of* CAPN1* gene are associated with meat quality and tenderness traits according to Shu et al.'s and Felício et al.'s researches [21, 22]. Simultaneously, several polymorphisms of* CAST* have also been described and identified to be associated with carcass quality and production traits [23, 24]. Among these markers, a SNP in the intron 5 of the bovine* CAST* gene, AY_008267.1:g282CNG, is associated with postmortem meat tenderness in crossbred* B. taurus* populations [25]. In 2010, Liu et al. found that a polymorphism (C36127T) of CAST gene has relation with carcass and tenderness traits in chickens [26]. Recently, Biswas et al. conducted an experiment to detect the change of expression of CAST in muscle of postmortem aging of meat during holding at refrigeration temperatures with the real-time PCR and found association between CAST gene and muscle change in chickens [27].

In our previous researches, we conducted association study of* CAPN1* and* CAST* with carcass and tenderness traits in the third generation of Da-Heng broilers. The results indicate that several SNPs in the exons or introns have significant effects on chicken carcass and tenderness traits [28, 29]. However, limited information about the change and clarification of SNPs and haplotypes after several generations is available. Is there any tendency or pattern? Therefore, to verify our hypothesis, we studied the association of* CAPN1* and* CAST* gene with carcass and tenderness traits in the ninth generation of Da-Heng broiler and conducted a comparison analysis between our previous studies in the third generation.

## 2. Materials and Methods

### 2.1. Chicken Population

The ninth generation of Da-Heng high-quality broilers, including five pure lines S01, S05, S06, S07, and S08 and the crossbred S01 × S08 line, were used in this study. Da-Heng high-quality broiler is a specialized meat type breeding with native chickens in Sichuan and Guangdong provinces of China by Sichuan Da-Heng Poultry Breeding Company. The selection focus of Da-Heng broiler breeding lines is different, such as growth rate and feed conversion rate. But the breeding of all the lines takes into account the carcass traits, and some takes into account the meat quality. Two-line crossbreeding S01 × S08 is a commercial crossbreed. Except for S01, each strain was randomly sampled with 30 male chickens and 30 female chickens for slaughtering and blood collecting. For S01, 35 male chickens and 35 female chickens were randomly sampled. Briefly, a total of 370 Da-Heng broilers of the ninth generation were sampled for sequencing.

### 2.2. Management and Slaughter Parameter Measurements

All chickens were fed based on the National Research Council's (NRC from 2014) requirements of broilers. At the age of 90 days, chickens were slaughtered by electric shock and blood samples were collected. Slaughter parameters including live weight (LW), carcass weight (CW), leg muscle weight (LMW), abdominal fat weight (AFW), skin fat thickness (SFT), breast muscle weight (BMW), semieviscerated weight (SEW), and eviscerated weight (EW) were measured according to the following description. The CW was measured on the chilled carcass after removal of feathers. SEW is a parameter of carcass weight of removal of the trachea, oesophagus, gastrointestinal tract, spleen, pancreas, and gonad. EW is a parameter of carcass weight of removal of all internal organs which was measured on the SEW after removal of the head, claws, heart, liver, gizzard, glandular stomach, and abdominal fat. The ratios of these traits to CW were calculated as eviscerated percentage (EP), semieviscerated percentage (SEP), breast muscle percentage (BMP), leg muscle percentage (LMP), and abdominal fat percentage (AFP). Tenderness was measured by Warner-Bratzler shear force (SF), which determines the relative force required to pass a blunt blade through a section of meat [30]. Water depletion rate (WDR) is drip losses rate measured according to the American Meat Science Association (AMSA) (1995) guidelines. All protocols used in this study were approved by Sichuan Agricultural University Animal Care and Use Committees.

### 2.3. DNA Extraction and PCR Amplification

Genomic DNA was extracted from the blood samples (15 *μ*l) by phenolic-chloroform method after digestion with proteinase K and precipitation with NaCl and alcohol.

The primer pairs CAST_SNP and CAPN1_SNP (Table 1) were designed by Primer Premier 5.0 according to the GenBank accession numbers NC_006127.4 and NT_464263.1, respectively. Cycling conditions consisted of an initial denaturation at 94°C for 6 min and 35 cycles at 94°C for 45 s, 48°C or 56°C (for CAST and CAPN1, resp.) for 45 s, and 72°C for 1 min, followed by a final step of 72°C for 8 min. PCR amplifications were performed in a total volume of 25 *μ*l containing 2.0 *μ*l (50 ng/*μ*l) of template, 12.5 *μ*l of 2x Taq PCR MasterMix, 8.5 *μ*l of ddH_2_O, and 1.0 *μ*l of each primer (10 pmol/*μ*l). PCR amplification product was detected by 1% agarose gel and gels were both visualized on Gel DocTMEQ170-8060 and photographed under UV light.

The PCR products were sequenced by TSINGKE Biological Technology Corporation, Sichuan, China.

### 2.4. Statistical Analysis

Data were analyzed by General Linear Model (GLM) procedures of SAS (SAS Inst. Inc., Cary, NC, USA). The line and genetic effects were analyzed by the following model: *Y* = *μ* + *L* + *S* + *G* + (*L* × *G*)+(*S* × *G*) + *e*, where *Y* is the traits measured on chickens, *μ* is the population mean, *e* is the random error, *L* is the fixed effect of line, *S* is the fixed effect of sex, *G* is the fixed effect associated with the genotype, (*L* × *G*) is the interaction between the line and genotype, and (*S* × *G*) is the interaction between the sex and genotype and it was excluded from the model if its value was *P* > 0.05 on a given trait. The values were presented as least square means ± standard error. The significance of least square means was tested with the Duncan test (*P* < 0.01 or *P* < 0.05).

## 3. Results

### 3.1. Detection of Polymorphisms in* CAPN1* and* CAST* Genes in the Ninth Generation

In this study (Table 3), a total of four polymorphisms were detected in* CAPN1* gene, including a G3535A (NC_006090.1:g.30419210G<A) transition in exon 6, a C7198A (NC_006090.1:g.30422873C<A) transition, a G7324A (NC_006090.1:g.30422999G<A) transition in exon 16, and a G9950A (NC_006090.1:g.30425625G<A) transition in intron 13. As for CAST gene, only a G37868A (NC_006127.2:g.57042952G<A) transition was found in intron 11.

Hardy-Weinberg equilibrium test of the 5 SNPs was shown in Table 3. As expected, all of the SNPs fitted the assumption of the Hardy-Weinberg equilibrium (*P* > 0.05) and the minor allele frequencies of all the mutations were more than 0.01. These results indicated that the observed heterozygosities of all SNPs in the ninth generations were at similar levels.

### 3.2. Genotype and Allele Frequencies in the Ninth Generations

The allele and genotype frequencies of the* CAPN1* and* CAST* obtained in different lines of the ninth generation were shown in Table 5. Chi-squared test suggested that SNP1 and SNP2 had significant correlations within each line, while SNP3 and SNP4 were not. For SNP1, SNP3, and SNP4, GG genotype was the favorable homozygote in most lines, and G was the advantageous allele. For SNP2, the frequency of allele C was higher than allele A, and CC genotype was the favorable one followed by AC and AA genotype. As for the SNP6 of* CAST*, genotype GG was rare and A is the advantageous allele.

### 3.3. Genetic Effect of* CAPN1* and* CAST* Gene on Carcass and Tenderness Traits

The results of association analysis in the ninth generation were summarized in Table 7. Our results showed that, in the ninth generation, SNP1 was significantly associated with BMW, SFT, SF (*P* < 0.05), and FT (*P* < 0.01) and indexes of genotype AG and GG were significantly higher than AA. SNP2 had a significant correlation with SEP and FT (*P* < 0.05) and AFW and AFP (*P* < 0.01), while genotype CC was significantly higher than the other two genotypes. SNP3 had a significant relation with LW, SEP, and SF (*P* < 0.05) and highly significant association with CW (*P* < 0.01); genotypes AA and AG were predominant for LW and CW. A significant association between SNP4 and LW, CW, BMW, and LMW (*P* < 0.05) was also observed, and indexes of homozygous GG were significantly higher. In* CAST*, there was a significant association between SNP5 and LW and CW (*P* < 0.05).

## 4. Discussion 

Both the carcass and tenderness traits are controlled by multiple genes [30]. Thus, understanding their genetic basis will promote the genetic improvement of the carcass and tenderness traits. The association analysis of candidate gene is one of the main methods to determine whether specific genes are associated with specific traits in economic animals.

Previous studies found that calpain system can improve muscle tenderness after slaughter by cleaved limited myofibrillar proteins such as titin, desmin, and vinculin, while high levels of* CAST* are related to decrease of proteolysis and increase of meat toughness [31]. Thus,* CAPN1* and* CAST *probably have significant effects on meat tenderness. In fact, the genotypic effects of* CAPN1* are able to significantly affect the carcass traits in many species [29, 32, 33]. However, reports about the effects of* CAST* on carcass traits instead of tenderness traits are rare in chickens.

The Hardy-Weinberg equilibrium is influenced by many factors, including selection, the rate of recombination, the rate of mutation, genetic drift, the system of mating, population structure, and genetic linkage. As expected, due to lack of foreign blood in current chicken population, the observed heterozygosity of all SNPs was at a similar level, and all SNPs fit the assumption of the Hardy-Weinberg equilibrium. Therefore, the five SNPs in the ninth generation are suitable for the association study.

Numerous studies report that calpain system plays important roles in carcass and tenderness traits [21, 34, 35]. In the present study, we examined* CAPN1* and* CAST* as two candidate genes for carcass and tenderness traits in the ninth generation of a commercial chicken breed and detected four SNPs in* CAPN1* and one SNP in* CAST *(Table 3). Compared with our previous research (Table 2), SNP3 in* CAPN1* was a novel mutation and had never been reported in previous studies. Our results suggested that SNP3 has a significant association with LW, CW, SEP, and SF. This may be due to genetic variation benefitting from meat quality breeding that resulted from specific environment and artificial selection during the breeding process. This mutation created a synonymous transition and may influence the transcription or translation of* CAPN1* gene. We did not detect A37752T in* CAST *in the ninth generation probably because of stronger artificial selection. In our previous study, there are significant associations between SNP5 and LMW (*P* < 0.05), CW, LW, and BMW (*P* < 0.01) in the third generation (Table 6). And we found individuals of genotype AA in SNP5 had significantly higher indexes than individuals of genotypes AT and TT. After intense artificial selection we reserved individuals of genotype AA and wiped out genotype AT and TT. This could be the reason of the miss of SNP5 in the ninth generation.

In order to investigate the genetic changes of* CAPN1* and* CAST *during the breeding process, we analyzed frequencies of alleles and genotypes of* CAPN1* and* CAST *in the third and the ninth generation. We found that all SNPs of* CAPN1* in the third generation were significantly associated within lines, while SNP4 and new site, SNP3, were not correlated with lines in the ninth generation. This indicated that difference of SNP4 in each line tended to reduce because of the lack of foreign blood. We found that the frequency of allele A in SNP2 of the ninth generation was higher than the third generation. It is interesting that the frequencies of allele in SNP6 in the two generations were extremely different. In the third generation, frequency of allele G was 0.540, slightly higher than allele A (Table 4). However, frequency of allele A in the ninth generation was 0.719, obviously higher than allele G. This situation could be explained by the combination of genetic effects of two generations. 

In the breeding process of Da-Heng broilers, we found that carcass indexes of the ninth generation were obviously higher than the third generation. In the third generation of Da-Heng broilers, we found that SNP1 had significant effect on BMP and SF and individuals of genotype GG had higher carcass indexes and better tenderness indexes, while, in ninth generation, SNP1 had significant association with BMW, SFT, SF, and WDR, and GG genotype still had higher carcass indexes and better tenderness traits. SNP2 in the third generation showed significant association with AFW and WDR. And there was significant correlation between SNP2 and SEP, AFW, AFP, and WDR in the ninth generation, and chickens of genotype CC had a higher AFW. The loss of genotype CC during the breeding process is probably because we need individuals with lower AFW. As a result, frequency of allele C in the ninth generation was lower than the third generation. SNP4 in the ninth generation had significant association with CW, LW, BMW, and LMW, and individuals of genotype GG had higher carcass indexes. This was similar to the third generation. These sites were relatively conservative during the breeding process. In the third generation, there was no association between SNP6 in* CAST* and carcass or tenderness traits, while SNP6 showed significant association with LW and CW in the ninth generation. Collectively, the association of SNP1 and SNP2 as well as SNP4 between carcass and tenderness traits was consistent in the two generations.

We found that carcass indexes and tenderness indexes in the ninth generation were higher than those in the third generation (Tables 6 and 7). And furthermore, individuals with better carcass and tenderness indexes have higher related genotype frequency. That is to say, the change of SNPs in these two genes has a direction of clarification under our breeding progress for meat quality. However, we did not conduct the comparison analysis with the original generation; this limited us from getting more information about the dynamic tendency of SNPs and haplotypes.

## 5. Conclusion 

In conclusion, the association between SNP1, SNP2, and SNP4 in CAPN1 gene with carcass and tenderness traits of different generations was very consistent. And these sites have slight changes toward the direction of benefitting meat type breeding under the pressure of artificial selection of broilers. We can use these consistent molecular markers for genetic markers assistant selection in chickens. We can also regard SNP3 of* CAPN1* as a potential molecular marker related to carcass and tenderness traits. In* CAST*, SNP5 might be a molecular marker for molecular assisted selection. However, the association between SNP6 and carcass as well as tenderness traits needs further verification. And the change of SNPs in the two genes has a direction of clarification during the six generations of breeding.

## Figures and Tables

**Table 1:** Primers for screening in the chicken *CAPN1* and *CAST* genes.

	Primers	Sequence of the primers	Amplified length	Binding regions	Tm (°C)
*CAPN1*	P1	F: TCA CCT CAC GTG CCT CTC TCA	217	Exon 5	58.0
		R: CGG AAC ACT TAC GTC GAT			
	P2	F: AGG GGT AGG GTA ATA GAA CTA	233	Exon 6	58.0
		R: ACC GCC AGC CAT CAA AT			
	P3	F: CCT CCT TCC TCC TCA GAC AAA	191	Exon 16	55.0
		R: CAGCCT TGG CAC AAC TAG AGA			
	P4	F: TCAGGACACTGG TGT TCA ATA	212	Intron 3	55.0
		R: GGA AAG GGT GTA GTG GTA C			

*CAST*	P5	F: AAT ACA GGG TCA CAT CG	239	Exon 8	56.0
		R: AAA GAA ACA TTC CCT GA			
	P6	F: AAA CGA GAA GGT AGC C	291	Exon 11	55.0
		R: CTG GTA TCT TTG GAA GAC ATA			
	P7	F: CCA AAA GTA GAT GAA CAT TCT	249	Intron 11	48.0
		R: GCT TCT ATT AAT TCC TAC CT			

**Table 2:** The Hardy-Weinberg equilibriumof *CAPN1 *and *CAST *gene mutation in the third generation.

Markers	Position	ObsHET	ExptHET	Allele change	Amino acids change	HWE (*P*)	MAF
SNP1	3535	0.360	0.328	G>A	215 Leu > Leu	0.094	0.371
SNP2	7198	0.462	0.370	C>A	427 Glu > His	0.185	0.683
SNP4	9950	0.637	0.396	G>A	932 Cys > Arg	0.751	0.339
SNP5	37752	0.368	0.853	A>T	307 Lys > Lys	0.484	0.454
SNP6	37868	0.621	0.376	G>A	335 Arg > His	0.962	0.588

*Notes*. ObsHET, observed heterozygosity; ExptHET, expected heterozygosity; HWE, Hardy-Weinberg equilibrium; MAF, minimum allele frequency.

**Table 3:** The Hardy-Weinberg equilibriumof *CAPN1 *and *CAST *gene mutation in the ninth generation.

Markers	Position	ObsHET	ExptHET	Allele change	Amino acids change	HWE (*P*)	MAF
SNP1	3535	0.439	0.5	G>A	215 Leu > Leu	0.150	0.488
SNP2	7198	0.396	0.44	C>A	427 Glu > His	0.262	0.326
SNP3	7324	0.488	0.473	G>A	489 Val > Val	0.850	0.384
SNP4	9950	0.53	0.496	G>A	932 Cys > Arg	0.357	0.639
SNP6	37868	0.488	0.498	G>A	335 Arg > His	0.882	0.470

*Notes*. ObsHET, observed heterozygosity; ExptHET, expected heterozygosity; HWE, Hardy-Weinberg equilibrium; MAF, minimum allele frequency.

**Table 4:** Genotype frequency and allele frequency of *CAPN1 *and *CAST *gene in the third generation.

Mutations	Lines	Frequency of genotypes			Frequency of alleles		*χ* ^2^, *P* value
		AA	GG	AG	A	G	
SNP1 G3535A	S08	0.00	0.00	1.00	0.50	0.50	*χ* ^2^ = 84.82, *P* = 0.0072
	S07	0.29	0.32	0.39	0.48	0.52	
	S06	0.00	0.25	0.75	0.37	0.63	
	S01	0.25	0.36	0.39	0.45	0.55	
	S05	0.26	0.41	0.33	0.43	0.57	
	S08 × S07	0.45	0.22	0.33	0.61	0.39	
Average value					0.473	0.527	

		AA	CC	AC	A	C	
SNP2 C7198A	S08	0.11	0.74	0.15	0.19	0.81	*χ* ^2^ = 32.52, *P* = 0.0341
	S07	0.07	0.57	0.36	0.25	0.75	
	S06	0.19	0.48	0.33	0.35	0.65	
	S01	0.08	0.71	0.21	0.18	0.82	
	S05	0.07	0.52	0.41	0.28	0.72	
	S08 × S07	0.11	0.68	0.21	0.21	0.79	
Average value					0.243	0.757	

		AA	GG	AG	A	G	
SNP4 G9950A	S08	0.07	0.85	0.08	0.11	0.89	*χ* ^2^ = 34.61, *P* = 0.0006
	S07	0.18	0.32	0.50	0.43	0.57	
	S06	0.185	0.185	0.63	0.50	0.50	
	S01	0.11	0.39	0.50	0.36	0.64	
	S05	0.00	0.00	1.00	0.50	0.50	
	S08 × S07	0.04	0.00	0.96	0.52	0.48	
Average value					0.403	0.597	

		AA	TT	AT	A	T	
SNP5 A33752T	S08	0.33	0.56	0.11	0.39	0.61	*χ* ^2^ = 48.44, *P* = 0.0009
	S07	0.07	0.57	0.36	0.25	0.75	
	S06	0.56	0.11	0.33	0.73	0.27	
	S01	0.25	0.25	0.50	0.50	0.50	
	S05	0.41	0.26	0.33	0.57	0.43	
	S08 × S07	0.36	0.56	0.08	0.40	0.60	
Average value					0.473	0.527	

		AA	GG	AG	A	G	
SNP6 A37868G	S08	0.18	0.30	0.52	0.44	0.56	*χ* ^2^ = 20.07, *P* = 0.3195
	S07	0.18	0.43	0.39	0.38	0.62	
	S06	0.15	0.41	0.44	0.37	0.63	
	S01	0.29	0.25	0.46	0.52	0.48	
	S05	0.26	0.26	0.48	0.50	0.50	
	S08 × S07	0.43	0.32	0.25	0.55	0.45	
Average value					0.460	0.540	

**Table 5:** Genotype frequency and allele frequency of *CAPN1 *and *CAST *gene in the ninth generation.

Mutations	Lines	Frequency of genotypes			Frequency of alleles		*χ* ^2^, *P* value
		AA	GG	AG	A	G	
SNP1 G3535A	S08 (60)	12 (0.2)	6 (0.1)	42 (0.7)	66 (0.55)	54 (0.45)	*χ* ^2^ = 17.34, *P* = 0.0006
	S07 (60)	18 (0.3)	21 (0.35)	21 (0.35)	57 (0.475)	63 (0.525)	
	S06 (60)	12 (0.2)	12 (0.2)	36 (0.6)	60 (0.5)	60 (0.5)	
	S01 (70)	20 (0.28)	21 (0.3)	29 (0.42)	69 (0.49)	71 (0.51)	
	S05 (60)	3 (0.05)	36 (0.6)	21 (0.35)	27 (0.225)	93 (0.775)	
	S08 × S07 (60)	33 (0.55)	12 (0.2)	15 (0.25)	81 (0.675)	39 (0.325)	
Average value					0.486	0.514	

		AA	CC	AC	A	C	
SNP2 C7198A	S08 (60)	6 (0.1)	36 (0.6)	18 (0.3)	30 (0.25)	90 (0.75)	*χ* ^2^ = 15.83, *P* = 0.0008
	S07 (60)	3 (0.05)	30 (0.5)	27 (0.45)	33 (0.275)	87 (0.725)	
	S06 (60)	21 (0.35)	18 (0.3)	21 (0.35)	63 (0.525)	57 (0.475)	
	S01 (70)	6 (0.094)	34 (0.484)	30 (0.422)	42 (0.305)	98 (0.695)	
	S05 (60)	9 (0.15)	12 (0.2)	59 (0.65)	57 (0.475)	63 (0.525)	
	S08 × S07 (60)	6 (0.1)	42 (0.7)	12 (0.2)	24 (0.2)	96 (0.8)	
Average value					0.338	0.662	

		AA	GG	AG	A	G	
SNP3 G7324A	S08 (60)	6 (0.1)	27 (0.45)	27 (0.45)	39 (0.325)	81 (0.675)	*χ* ^2^ = 1.75, *P* = 0.0735
	S07 (60)	6 (0.1)	21 (0.35)	33 (0.55)	45 (0.375)	75 (0.625)	
	S06 (60)	9 (0.15)	24 (0.4)	27 (0.45)	45 (0.375)	75 (0.625)	
	S01 (70)	5 (0.078)	27 (0.391)	38 (0.531)	48 (0.3429)	92 (0.6571)	
	S05 (60)	3 (0.05)	30 (0.5)	27 (0.45)	33 (0.275)	87 (0.725)	
	S08 × S07 (60)	12 (0.2)	24 (0.4)	24 (0.4)	48 (0.4)	72 (0.6)	
Average value					0.357	0.643	

		AA	GG	AG	A	G	
SNP4 G9950A	S08 (60)	6 (0.1)	30 (0.5)	24 (0.4)	36 (0.3)	84 (0.7)	*χ* ^2^ = 7.60, *P* = 0.1320
	S07 (60)	6 (0.1)	15 (0.25)	39 (0.65)	51 (0.425)	69 (0.575)	
	S06 (60)	15 (0.25)	18 (0.3)	27 (0.45)	57 (0.475)	63 (0.525)	
	S01 (70)	16 (0.234)	13 (0.188)	41 (0.578)	73 (0.523)	67 (0.477)	
	S05 (60)	12 (0.2)	15 (0.25)	33 (0.55)	57 (0.475)	63 (0.525)	
	S08 × S07 (60)	9 (0.15)	24 (0.4)	27 (0.45)	45 (0.375)	75 (0.625)	
Average value					0.429	0.571	

		AA	GG	AG	A	G	
SNP6 A37868G	S08 (60)	45 (0.75)	0 (0.0)	15 (0.25)	105 (0.875)	15 (0.125)	*χ* ^2^ = 8.07, *P* = 0.0431
	S07 (60)	33 (0.55)	0 (0.0)	27 (0.45)	93 (0.775)	27 (0.225)	
	S06 (60)	21 (0.35)	3 (0.05)	36 (0.6)	78 (0.65)	42 (0.35)	
	S01 (70)	30 (0.429)	3 (0.043)	37 (0.528)	97 (0.6929)	43 (0.3071)	
	S05 (60)	24 (0.4)	6 (0.1)	30 (0.5)	78 (0.65)	42 (0.35)	
	S08 × S07 (60)	27 (0.45)	6 (0.1)	27 (0.45)	81 (0.675)	39 (0.325)	
Average value					0.719	0.281	

**Table 6:** Genetic effect of *CAPN1 *and* CAST* gene on carcass traits and tenderness traits in the third generation of Da-Heng broiler.

Polymorphism	Genotype	Indexes												
		LW (g)	CW (g)	SEP (%)	EP (%)	BMW (g)	BMP (%)	LMW (g)	LMP (%)	AFW (g)	AFP (%)	SFT (mm)	SF (Kg·N)	WDR (%)
SNP1	AA 75	1636.57 ± 44.44	1505.11 ± 49.25	88.18 ± 0.36	72.79 ± 0.36	170.48 ± 5.79	13.21 ± 0.20^b^	255.28 ± 9.26	19.35 ± 0.25	32.44 ± 2.72	2.45 ± 0.20	3.79 ± 0.29	2.86 ± 0.12^a^	73.06 ± 0.98
	AG 199	1636.14 ± 42.07	1471.85 ± 46.59	88.18 ± 0.34	73.13 ± 0.34	174.31 ± 5.48	13.51 ± 0.19^a^	249.53 ± 9.76	19.21 ± 0.24	36.10 ± 2.57	2.62 ± 0.19	3.80±0.31	2.58±0.16b	75.89±1.31
	GG 96	1561.89 ± 46.33	1413.32 ± 51.34	88.40 ± 0.38	73.16 ± 0.38	171.54 ± 6.04	13.83 ± 0.21^a^	246.15 ± 9.66	19.51 ± 0.27	33.94 ± 2.84	2.73 ± 0.21	3.85 ± 0.26	3.01 ± 0.15^b^	74.38 ± 1.19

SNP2	AA 38	1596.73 ± 59.07	1499.69 ± 65.46	88.34 ± 0.49	73.46 ± 0.48	174.63 ± 7.70	13.73 ± 0.27	248.77 ± 12.32	19.41 ± 0.34	35.19 ± 3.62^a^	2.77 ± 0.26	3.72 ± 0.36	2.90 ± 0.17	72.76 ± 1.33^b^
	AC 103	1625.57 ± 42.87	1448.87 ± 47.51	88.20 ± 0.35	72.60 ± 0.35	171.56 ± 5.59	13.41 ± 0.19	251.64 ± 8.94	19.36 ± 0.24	35.45 ± 2.62^a^	2.59 ± 0.19	3.57 ± 0.29	2.83 ± 0.15	75.16 ± 1.21^a^
	CC 229	1612.30 ± 35.09	1441.72 ± 38.89	88.21 ± 0.29	73.02 ± 0.28	170.14 ± 4.57	13.41 ± 0.16	250.56 ± 7.31	19.30 ± 0.20	37.84 ± 2.15^b^	2.87 ± 0.15	3.85 ± 0.40	2.71 ± 0.13	75.41 ± 0.89^a^

SNP4	AA 37	1537.19 ± 45.61^b^	1376.14 ± 50.54^B^	88.19 ± 0.48	72.42 ± 0.48	165.84 ± 5.95	13.41 ± 0.27	237.71 ± 9.51	19.17 ± 0.26^b^	34.21 ± 3.60	2.49 ± 0.26	3.89 ± 0.32	2.66 ± 0.19	74.64 ± 1.48
	AG 225	1585.28 ± 38.48^b^	1416.65 ± 42.65^B^	88.04 ± 0.32	73.05 ± 0.31	168.81 ± 5.02	13.43 ± 0.17	243.39 ± 8.02	18.90 ± 0.22^b^	35.34 ± 2.36	2.69 ± 0.17	3.97 ± 0.27	2.94 ± 0.14	73.57 ± 1.10
	GG 108	1712.13 ± 58.77^a^	1597.49 ± 65.12^A^	88.51 ± 0.37	73.62 ± 0.37	181.68 ± 7.66	13.71 ± 0.21	269.87 ± 12.25	19.91 ± 0.34^a^	32.93 ± 2.79	2.62 ± 0.20	3.71 ± 0.33	2.84 ± 0.12	75.13 ± 0.99

SNP5	AA 122	1673.50 ± 34.89^A^	1519.33 ± 38.71^A^	88.84 ± 0.29	73.22 ± 0.29	179.90 ± 4.51^A^	13.54 ± 0.16	253.30 ± 7.31^a^	18.80 ± 0.20	35.04 ± 2.15	2.56 ± 0.15	4.16 ± 0.30	2.79 ± 0.14	73.25 ± 1.17
	AT 143	1507.26 ± 32.53^B^	1337.72 ± 36.09^B^	88.18 ± 0.27	72.71 ± 0.27	160.66 ± 4.20^B^	13.49 ± 0.15	229.29 ± 6.81^b^	18.94 ± 0.19	33.37 ± 2.00	2.70 ± 0.14	3.89 ± 0.25	2.94 ± 0.11	74.32 ± 0.93
	TT 105	1578.90 ± 34.87^B^	1398.64 ± 38.69^B^	88.96 ± 0.29	73.60 ± 0.29	166.48 ± 4.50^B^	13.33 ± 0.16	243.39 ± 7.30^b^	19.03 ± 0.20	31.47 ± 2.14	2.34 ± 0.16	4.13 ± 0.34	2.61 ± 0.13	74.49 ± 1.08

SNP6	AA 92	1611.94 ± 36.73	1472.52 ± 40.46	88.79 ± 0.30	72.92 ± 0.30	170.45 ± 4.74	13.29 ± 0.17	247.20 ± 7.69	19.05 ± 0.21	34.91 ± 2.26	2.65 ± 0.16	3.93 ± 0.28	2.92 ± 0.15	73.65 ± 1.19
	AG 156	1557.93 ± 32.50	1373.32 ± 36.06	88.77 ± 0.27	73.22 ± 0.27	167.46 ± 4.20	13.64 ± 0.15	236.24 ± 6.80	18.77 ± 0.19	31.00 ± 2.00	2.37 ± 0.14	4.04 ± 0.27	2.73 ± 0.12	74.75 ± 0.96
	GG 122	1589.78 ± 32.18	1409.86 ± 35.70	88.42 ± 0.26	73.38 ± 0.26	169.12 ± 4.15	13.44 ± 0.15	242.54 ± 6.74	18.95 ± 0.18	33.96 ± 1.98	2.57 ± 0.14	3.86 ± 0.29	2.79 ± 0.13	73.64 ± 1.03

*Note*. Values with different letters in the same row differ significantly; the lowercase letters show the level of 0.05; the capital letters show the level of 0.01. LW, live weight; CW, carcass weight; SEP, semieviscerated percentage; EP, eviscerated percentage; BMW, breast muscle weight; BMP, breast muscle percentage; LMW, leg muscle weight; LMP, leg muscle percentage; AFW, abdominal fat weight; AFP, abdominal fat percentage; SFT, skin fat thickness; SF, shear force; WRD, water depletion rate.

**Table 7:** Genetic effect of *CAPN1 *and* CAST* gene on carcass traits and tenderness traits in the ninth generation of Da-Heng broiler.

Polymorphism	Genotype	Indexes												
		LW (g)	CW (g)	SEP (%)	EP (%)	BMW (g)	BMP (%)	LMW (g)	LMP (%)	AFW (g)	AFP (%)	SFT (mm)	SF (Kg·N)	WDR (%)
SNP1	AA 98	2319.65 ± 172.46	2114.60 ± 166.91	83.10 ± 1.02	71.08 ± 1.09	130.67 ± 12.77^a^	15.85 ± 0.89	171.97 ± 17.79	20.78 ± 1.10	26.03 ± 9.32	1.20 ± 0.45	3.31 ± 0.23^a^	2.46 ± 0.09^a^	70.83 ± 0.77^A^
	AG 164	2490.39 ± 191.23	2298.50 ± 185.08	83.85 ± 1.13	72.21 ± 1.20	145.21 ± 14.16^b^	16.03 ± 0.99	187.18 ± 19.73	20.61 ± 1.22	40.31 ± 10.34	1.65 ± 0.51	4.03 ± 0.26^b^	2.68 ± 0.13^b^	74.80 ± 1.07^B^
	GG 108	2677.54 ± 247.55	2466.78 ± 239.58	83.38 ± 1.47	73.10 ± 1.56	154.76 ± 18.33^b^	15.68 ± 1.28	200.61 ± 25.54	20.20 ± 1.58	36.34 ± 13.38	1.36 ± 0.65	4.22 ± 0.33^b^	2.81 ± 0.17^b^	74.72 ± 1.82^B^

SNP2	AA 52	2589.96 ± 308.29	2389.15 ± 298.37	82.15 ± 1.83^a^	70.53 ± 1.94	156.23 ± 22.82	16.81 ± 1.59	184.01 ± 31.81	19.93 ± 1.97	21.44 ± 16.67^A^	0.77 ± 0.81^A^	3.92 ± 0.41	2.90 ± 0.17	72.76 ± 1.33^a^
	AC 146	2433.38 ± 207.17	2226.08 ± 200.50	83.96 ± 1.23^a^	73.31 ± 1.30	134.81 ± 15.34	15.12 ± 1.07	181.83 ± 21.38	20.07 ± 1.32	37.03 ± 11.20^B^	1.59 ± 0.55^B^	3.78 ± 0.28	2.83 ± 0.15	75.16 ± 1.21^b^
	CC 172	2464.24 ± 153.59	2264.66 ± 148.65	84.22 ± 0.91^b^	72.56 ± 0.97	139.59 ± 11.37	15.63 ± 0.79	193.90 ± 15.84	21.58 ± 0.98	44.21 ± 8.30^B^	1.85 ± 0.41^B^	3.86 ± 0.21	2.71 ± 0.13	75.41 ± 0.89^b^

SNP3	AA 45	2645.81 ± 239.01^a^	2444.43 ± 231.32^A^	82.71 ± 1.42^a^	71.60 ± 1.50	147.41 ± 17.69	15.40 ± 1.24	194.77 ± 24.66	20.2 ± 1.53	24.99 ± 12.92	0.90 ± 0.63	3.92 ± 0.32	2.86 ± 0.12^a^	73.06 ± 0.98
	AG 171	2636.26 ± 228.84^a^	2424.47 ± 221.48^A^	83.40 ± 1.35^ab^	72.64 ± 1.44	157.29 ± 16.95	16.43 ± 1.18	192.09 ± 23.61	19.70 ± 1.46	39.90 ± 12.37	1.54 ± 0.60	4.01 ± 0.31	2.58 ± 0.16^a^	75.89 ± 1.31
	GG 154	2205.52 ± 181.97^b^	2010.98 ± 176.12^B^	84.22 ± 1.08^b^	72.15 ± 1.14	125.92 ± 13.47	15.72 ± 0.94	172.88 ± 18.78	21.61 ± 1.16	37.79 ± 9.84	1.77 ± 0.48	3.64 ± 0.24	3.01 ± 0.15^b^	74.38 ± 1.19

SNP4	AA 64	2294.19 ± 212.75^a^	2093.88 ± 205.91^a^	82.82 ± 1.26	71.43 ± 1.34	133.77 ± 15.75^a^	16.21 ± 1.10	166.33 ± 21.95^a^	20.22 ± 1.36	37.51 ± 11.50	1.74 ± 0.56	4.20 ± 0.29	2.66 ± 0.19	74.64 ± 1.48
	AG 190	2425.16 ± 157.86^a^	2227.42 ± 152.78^a^	83.56 ± 0.93	71.44 ± 0.99	129.84 ± 11.69^a^	14.90 ± 0.82	180.05 ± 16.29^a^	20.64 ± 1.01	24.67 ± 8.53	1.02 ± 0.42	3.36 ± 0.21	2.94 ± 0.14	73.57 ± 1.10
	GG 116	2768.23 ± 268.10^b^	2558.58 ± 259.47^b^	83.95 ± 1.59	73.53 ± 1.69	167.02 ± 19.85^b^	16.45 ± 1.39	213.37 ± 27.66^b^	20.72 ± 1.71	40.50 ± 14.49	1.45 ± 0.71	4.01 ± 0.36	2.84 ± 0.12	75.13 ± 0.99

SNP6	AA 180	2634.78 ± 189.73^a^	2441.58 ± 199.93^a^	83.42 ± 1.32	73.18 ± 1.34	137.96 ± 16.17	14.31 ± 0.98	193.97 ± 25.48	20.12 ± 1.17	32.67 ± 9.83	1.24 ± 0.39	4.38 ± 0.21	2.92 ± 0.15	73.65 ± 1.19
	AG 171	2590.48 ± 203.95^a^	2383.39 ± 197.38^a^	83.40 ± 1.21	72.33 ± 1.28	143.10 ± 15.10	14.97 ± 1.05	194.39 ± 21.04	20.51 ± 1.30	34.70 ± 11.03	1.34 ± 0.54	4.15 ± 0.27	2.73 ± 0.12	74.75 ± 0.96
	GG 19	2401.24 ± 158.37^b^	2203.19 ± 153.28^b^	83.49 ± 0.94	71.93 ± 1.00	143.98 ± 11.72	16.73 ± 0.82	178.78 ± 16.34	20.22 ± 1.01	33.76 ± 8.56	1.47 ± 0.42	3.56 ± 0.21	2.79 ± 0.13	73.64 ± 1.03

*Note*. Values with different letters in the same row differ significantly; the lowercase letters show the level of 0.05; the capital letters show the level of 0.01. LW, live weight; CW, carcass weight; SEP, semieviscerated percentage; EP, eviscerated percentage; BMW, breast muscle weight; BMP, breast muscle percentage; LMW, leg muscle weight; LMP, leg muscle percentage; AFW, abdominal fat weight; AFP, abdominal fat percentage; SFT, skin fat thickness; SF, shear force; WRD, water depletion rate.
